# Syrian Refugee and Turkish Women with Breast Cancer: A Comparison on Clinicopathological Features and Survival

**DOI:** 10.34172/aim.2023.05

**Published:** 2023-01-01

**Authors:** Elif Atag, Serkan Gokcay, Eda Tanrikulu Simsek, Fatih Aslan, Abdullah Evren Yetisir, Murat Sari

**Affiliations:** ^1^Department of Medical Oncology, Haydarpasa Numune Training and Research Hospital, Istanbul, Turkey; ^2^Deparment of Medical Oncology, Private Silivri Anadolu Hospital, Istanbul, Turkey; ^3^Department of General Surgery, Sanliurfa Mehmet Akif Inan Training and Research Hospital, Sanliurfa, Turkey; ^4^Department of Medical Oncology, Adana City Training and Research Hospital, Adana, Turkey

**Keywords:** Breast cancer, Chemotherapy, Survival, Syrian refugees, Vulnerable people, Women

## Abstract

**Background:**

Cancer is a significant health problem for refugees and host countries. Breast cancer is the most common cancer among refugees. The subject of our study is to examine the clinical and pathological features of Syrian refugees with breast cancer and compare them with Turkish patients with breast cancer.

**Methods:**

Data of patients with breast cancer between January 2018 and December 2020 were retrospectively reviewed. The clinical and histological features, treatment modalities and overall survival were collected and analyzed.

**Results:**

A total number of 338 women with breast cancer were included in this study. Ninety-nine of the 338 (29.3%) patients were Syrian refugees and 239 patients (70.7%) were Turkish. The median follow-up time was significantly lower in Syrian patients (*P*<0.001). Median OS was 146 months in Turkish and 116 months in Syrian group (*P*=0.022). Independent risk factors associated with long survival were receiving adjuvant chemotherapy (HR 0.465; 95% CI 0.234–0.926; *P*=0.029), adjuvant radiotherapy (HR 0.372 95% CI 0.182–0.758; *P*=0.007), and adjuvant hormonotherapy (HR 0.367; 95% CI 0.201–0.669; *P*=0.001). The rates of receiving adjuvant chemotherapy, adjuvant radiotherapy, and adjuvant hormonal therapy were significantly lower in the Syrian group (*P*=0.023, *P*=0.005, *P*=0.002, respectively).

**Conclusion:**

Syrian refugees with breast cancer are more likely to receive suboptimal treatments. They have inferior survival compared to local patients. Our findings highlight the need for the provision of cancer therapy in such vulnerable populations. We suggest that more attention should be paid to breast cancer, as it is the most common cancer among refugees.

## Introduction

 Since the beginning of the civil war in Syria in 2011, over 6.6 million people have taken refuge in more than 130 countries. Approximately 5.6 million refugees live in neighboring countries within the region, such as Turkey, Lebanon, Jordan, Iraq, and Egypt. European countries host over 1 million Syrian asylum-seekers and refugees, with 70% being hosted in only two countries: Germany (59%) and Sweden (11%).^1^ Turkey continues to host the largest number of refugees worldwide and currently hosts some 3.6 million registered Syrian refugees.^2^ The Syrian crisis was a global humanitarian crisis that concerned not only the region but also other countries. Refugees represent a highly vulnerable population; they require specific support and a major responsibility on the international community and host countries to meet their needs. Humanitarian crises always include medical crises and healthcare is a crucial part of this support and responsibility.

 In addition to communicable diseases, chronic non-communicable diseases are also now recognized as health problems that need to be addressed in such crises. Cancer in particular, is not only a leading cause of mortality but requires a healthcare system that can deliver multidisciplinary care and a wide range of services, such as diagnosis, palliation, and treatment with surgery, radiotherapy (RT), and chemotherapy (CT). Challenging situations such as conflict, war and migration can negatively affect cancer outcomes by causing delays in cancer diagnosis and inability to access treatments for patients.^3-5^ Data from studies showed that breast cancer (BC) is the most common type of cancer among all Syrian refugees.^6-8^

 BC is the most common malignancy diagnosed among women in the world, accounting for 24% of all new cancer cases. It is also the most common cause of cancer death in females worldwide.^9^ It is a potentially curable disease, especially in the early stage, and there has been substantial progress with new treatment agents even in metastatic disease. Very limited data showed that Syrian refugees with BC present late, have more advanced-stage disease and are more likely to receive delayed and suboptimal therapy.^5^ We did not find any data showing the survival of refugees with BC.

 The subject of our study is to examine the clinical and pathological features of Syrian refugees with BC who were followed up and/or treated at our center. We also aimed to compare the survival of Syrian refugees and Turkish women with BC and find out the probable factors affecting outcomes during the crisis.

## Material and Methods

 Data of patients with BC treated at the University of Health Sciences, Sanliurfa Mehmet Akif Inan Training and Research Hospital between January 2018 and December 2020 were retrospectively reviewed after local ethical committee approval was obtained. All data were recorded from a hospital-based electronic health information system.

 The inclusion criteria:

- Patients who were Syrian and Turkish women with invasive BC (including patients diagnosed in Syria)
- Patients were 18 years or older in age.
- The exclusion criteria:
- Patients with *in situ* tumor
- Patients who had fewer than three medical encounters in our center.

Data on age at diagnosis, stage, menopausal status, histological features of the tumor, clinical subtype, treatment modalities including surgery, adjuvant CT, neoadjuvant CT, adjuvant hormonotherapy (HT), adjuvant and palliative RT, relapse or progression status and outcome were collected and analyzed.

 Staging was performed according to the American Joint Committee on Cancer anatomic stage groups 8th edition.^10^ In survival analyses, stages 1 and 2 were grouped as early-stage disease, stage 3 was locally advanced disease, and stage 4 was metastatic disease. Relapse was defined as new evidence of disease after attaining remission. Progression was defined as detection of new clinical or radiological findings or worsening findings that were present at diagnosis during therapy. Outcomes were recorded as alive or dead. Follow-up time was determined as the time from diagnosis to the last visit or death. In the follow-up period, metastatic patients were evaluated every 3 months with physical examination, symptom questioning, laboratory test including tumor markers and appropriate imaging methods. Early-stage patients who underwent surgery were evaluated every 3 months for the first 2 years, every 6 months for the years 2–5, and once a year after the 5th year by physical examination, symptom questioning, laboratory test including tumor markers and necessary imaging methods. Patients who could not come to regular follow-up were re-evaluated at each visit and encouraged to adapt to the follow-up.

 Estrogen receptor (ER), progesterone receptor (PR), and human epidermal growth factor receptor-2 (HER2) status was evaluated using recommendations from the American Society of Clinical Oncology/College of American Pathologists.^11,12^ Using immunohistochemistry, cerbb2 0 and 1 + tumors were grouped as HER2 negative, 3 + tumors grouped as HER2 positive, and for cerbb2 2 + tumors, FISH (fluorescence *in situ* hybridization) was performed to confirm HER2 status. Patients without any data on HER2 status were defined as unknown.

 The patients were divided into three clinical subtypes as follows: Hormone receptor-positive (ER + , HER2 -), HER2 positive (HER2 + and ER + /-), and triple-negative (ER -, PR -, HER2).

###  Statistical Analysis

 IBM SPSS Statistics version 22.0 (SPSS, Inc., Chicago, IL, USA) was used for statistical analysis. Demographic variables were evaluated with descriptive statistics. The Chi-square test was used for the comparison of categorical measurements between groups. Mann-Whitney U and student *t* test were used to compare two groups of numerical variables according to the distribution of variables. The Kaplan-Meier method was used for survival analysis and a log-rank test was performed to compare survival in different groups. Overall survival (OS) was defined as the duration from the date of diagnosis to death or last follow-up, with no restriction on the cause of death. Univariate and multivariate analysis were performed to determine the effect of independent risk factors on prognosis using the Cox-regression method which was adjusted for adjuvant HT, radiotherapy, chemotherapy, nationality and stage at diagnosis. A *P *value less than 0.05 was considered as statistically significant.

## Results

 A total number of 338 women with BC were eligible and included in this study. The median age was 46 years (age range: 19–104). Ninety-nine of the 338 (29.3%) patients were Syrian refugees and 239 patients (70.7%) were Turkish. The clinicopathological characteristics and treatment modalities of Turkish and Syrian patients are given in Table 1. Ninety-seven of 99 Syrian patients were diagnosed in 2011 and later.

**Table 1:** Comparison of the Clinicopathological Characteristics and Treatment Modalities of Turkish and Syrian Patients

**Characteristics**	**All Patients** **(N=338)**	**Turkish Patients** **(n=239)**	**Syrian Patients** **(n=99)**	***P *** **Value**
Median age, years (Age range)	46 (19–104)	46 (27–86)	44 (19–104)	> 0.05
Menopausal status (%)				
Premenopausal	56.4	54.8	60.6	
Postmenopausal	34	36	29.3	0.498
Premenopausal	9.5	9.2	10.1	
Stage of tumor at diagnosis (%)				0.192
Stage 1	6.2	6.7	5.1	
Stage 2	26.6	29.7	19.2	
Stage 3	39.6	38.9	41.4	
Stage 4	15.7	14.6	18.2	
Unknown*	11.8	10	16.2	
Histology (%)				0.456
Ductal	68	66.5	71.7	
Lobular	8.3	7.9	9.1	
Other	13.8	14.6	11.1	
Unknown*	10.1	10.9	8.1	
Estrogen receptor (%)				0.515
Positive	74	75.7	69.7	
Negative	24.6	23.8	26.3	
Unknown*	1.5	0.4	4	
Progesterone receptor (%)				0.163
Positive	66.9	70.7	57.6	
Negative	29.9	28.5	33.3	
Unknown*	3.3	0.8	9.1	
HER2 (%)				0.639
Positive	30.5	32.2	26.3	
Negative	65.1	66.5	61.6	
Unknown*	4.4	1.3	12.1	
Tumor size (%)				0.015**
< 50 mm	52.7	57.3	41.4	
≥ 50 mm	27.5	23.4	37.4	
Unknown*	19.8	19.2	21.2	
Axillary stage (%)				0.323
N0	17.3	19.2	12.4	
N1	25	23	29.9	
≥ N2	35.7	36.4	34	
Unknown*	22	21.3	23.7	
Clinical subtype (%)				0.624
Hormone positive	54.7	56.5	50.5	
HER-2 positive	30.5	32.2	26.3	
Triple negative	11.5	10.9	13.1	
Unknown*	3.3	0.4	10.1	
Surgery				0.148
Lumpectomy	18.6	20.1	15.2	
Mastectomy	66.9	68.6	62.6	
Unknown	3	1.7	6.1	
No surgery	11.5	9.6	16.2	
Adjuvant CT (yes)	61.8	65.7	52.5	0.023
Neoadjuvant CT (yes)	14.8	14.6	15.2	0.905
Adjuvant HT (yes)	62.1	67.4	49.5	0.002
Site of metastasis (%)				
Local (breast or chest wall)	4.4	2.9	8.1	0.036
Bone	24.3	25.1	22.2	0.574
Lung and/or pleura	16.3	16.3	16.2	0.972
Liver	8.6	6.7	13.1	0.054
Brain	5.9	3.8	11.1	0.009
Other	4.7	2.9	9.1	0.015

HER-2: Human epidermal growth factor receptor-2, CT: Chemotherapy, RT: Radiotherapy, HT: Hormonotherapy. * No information or not evaluated. ** Statistical significance remains when the unknown is excluded (*P*= 0.004)

 The median follow-up time was 42 months (Range: 1–252 months) in all patients, 33 months (Range: 1–252) in Syrians and 47 months (Range: 8–158 months) in Turks (*P*< 0.001). Relapse or progression was detected in 80 (33.5%) Turkish patients and 43 (43.4%) in the Syrian group (*P*= 0.083).

**Figure 1:**
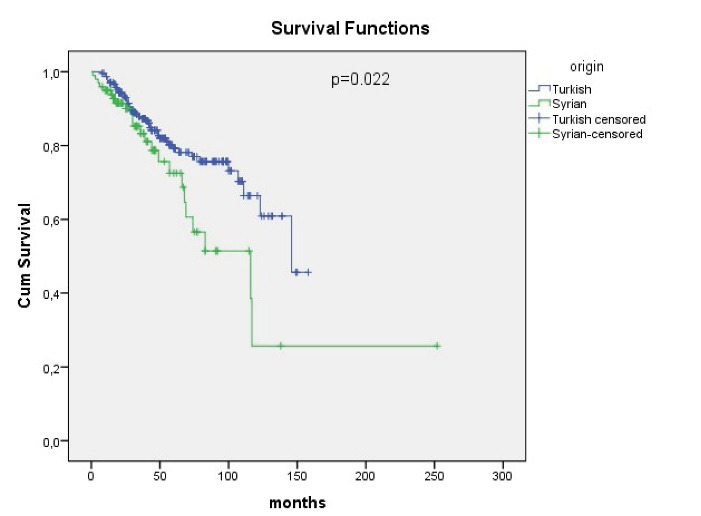
Overall Survival in Turkish and Syrian Patients.

**Figure 2:**
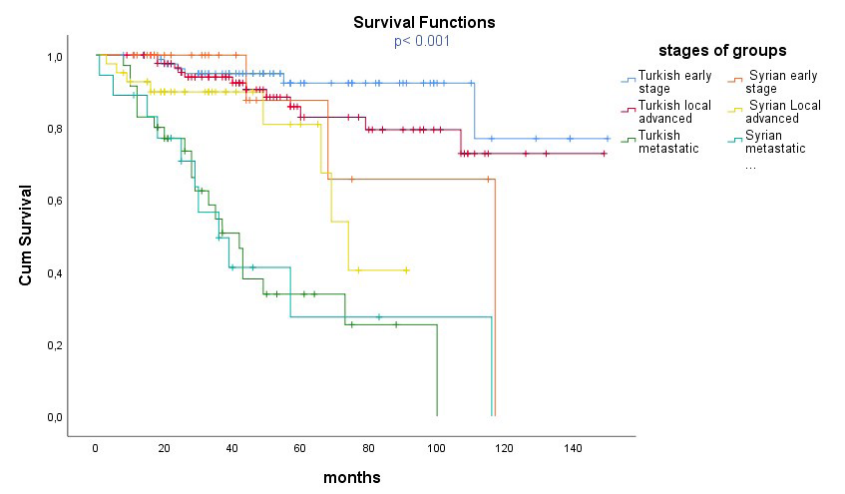
Overall Survival of Groups According to Tumor Stage.

 At the end of the study period, 80.8% of Turkish patients and 75.8% of Syrian patients were alive. Median OS was 146 months in Turkish patients and 116 months in Syrian patients (*P*= 0.022) (Figure 1). The 3-year OS was 87% in the Turkish group and 83% in the Syrian group, whereas the 5-year OS was 79% in the Turkish group and 72% in the Syrian group. Among patients with early-stage, median OS was not reached in Turkish and 117 months in Syrian (*P*= 0.165). In patients with locally advanced stage, median OS was not reached in Turkish and 74 months in Syrian (*P*= 0.026). In patients with metastatic disease, median OS was 42 months in Turks and 36 months in Syrians (*P*= 0.944) (Figure 2).

**Figure 3:**
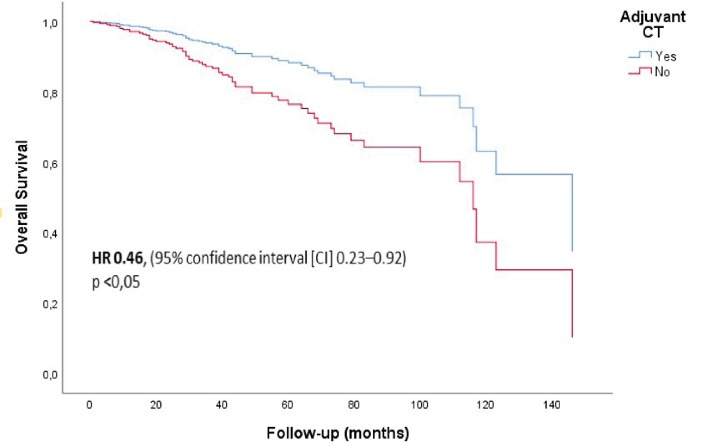
Overall Survival of Patients Who Received and Did Not Receive Adjuvant CT.

 In Cox regression analysis by creating a model with adjuvant CT, adjuvant RT, adjuvant HT, initial stage and nationality, the independent risk factors associated with long survival were receiving adjuvant CT (HR 0.465; 95% confidence interval [CI] 0.234–0.926; *P*= 0.029) (Figure 3), adjuvant RT (HR 0.372 95% CI 0.182–0.758; *P*= 0.007), and adjuvant HT (HR 0.367; 95% CI 0.201–0.669; *P*= 0.001) (Table 2).

**Table 2:** Univariate and Multivariate Cox Regression Analysis for Survival

	**Univariate**			**Multivariate**		
	**HR**	**95%CI**	***P***	**HR**	**95%CI**	***P***
Adjuvant HT ( + )	0.246	0.150–0.403	< 0.001	0.367	0.201–0.669	0.001
Adjuvant RT ( + )	0.108	0.044–0.262	< 0.001	0.372	0.182–0.738	0.007
Adjuvant CT ( + )	0.261	0.157–0.434	< 0.001	0.465	0.234–0.926	0.029
Nationality, Syrian	1.841	1.119–3.029	0.016			
Stage at diagnosis, metastatic	7.698	3.431–17.271	< 0.001			

## Discussion

 In the Syrian crisis, which has turned into a global problem, thousands of people have suffered due to health problems. Patients with cancer who had already a serious disease also experienced this chaotic period. Women with BC were a significant part of these patients. We believe that highlighting this important health problem is critical for the planning of healthcare for BC among refugees. Cancer among the Syrian refugees also represents a substantial financial burden for host countries and humanitarian agencies.^13^ Considering that BC can be cured at high rates with early detection and treatment, establishing screening programs for refugees and providing access to treatment will increase survival and significantly reduce the economic burden of cancer.

 The clinical features of Turkish and Syrian patients were similar. Only the rate of tumor size greater than 5 cm was significantly higher in the Syrian group. It may be related to the delayed finding of the breast mass by Syrian women or their delayed referral to professional healthcare services despite noticing it. The rates of initial tumor stages were not different between the groups, although numerically, the rates of the early stage were higher in Turkish patients, and the rates of the locally advanced and metastatic stage were higher in Syrian patients. In addition, 16.2% of Syrian patients and 10% of Turkish patients had an unknown initial stage. The unknown stages at the time of diagnosis and the small number of Syrian patients may have been effective in not showing the late presentation that we expect because of difficulties they face and the living conditions of refugees.

 The initial stages of both groups were more advanced compared to Western data.^14^ We believe that this may be related to the low socioeconomic level of both populations where the study was conducted. The lower socioeconomic level was associated with the late-stage diagnosis of BC.^15,16^

 Prognostic factors other than the stage in BC are ER and PR status, HER2 overexpression, Ki-67 proliferation index, and tumor grade, which are defined as biological prognostic factors.^9^ In our study, Ki-67 index and the grade of the tumor could not be evaluated because of the missing data in more than 50% of the patients. The clinical subtypes based on biological prognostic factors and histopathological features of the tumor were similar in Turkish and Syrian patients. This may be related to the fact that the place where the study was carried out is a border city to Syria and the people living in this city commonly have similar ethnic origins with refugees.

 In our study, the rates of receiving adjuvant CT, adjuvant HT, and adjuvant RT were significantly lower in the Syrian group, although they had similar clinical subtypes and stages compared to the Turkish group. Once refugees are registered and an identification number is given, they can access all healthcare facilities without any payment in Turkey. Our center is a public hospital and offers cancer treatments to refugees free of charge, including more expensive treatments such as trastuzumab. Therefore, the reason for receiving these treatments at lower rates was not lack of health insurance. One of the reasons may be their low adherence to the treatment in Turkey. We did not evaluate the treatment compliance in this study. However, another study conducted in the same region showed that the treatment compliance of Syrians was low.^7^ The living conditions and language-related communication problems of Syrians may have led to non-compliance to the treatments. In addition, patients who were diagnosed in Syria probably could not access the adjuvant treatments. On the other hand, we did not know which agents were used in patients who received adjuvant CT in Syria. These patients might have received incomplete treatments.

 Endocrine therapy reduces the risk of systemic recurrence and death among women with hormone receptor-positive BC, regardless of age, menopausal status, nodal involvement, tumor size, HER2 status, or use of CT.^9^ Although endocrine therapies are oral drugs that are easy to use and not costly, the inability of patients to receive this treatment may reflect their lack of follow-up. RT is an integral part of the multidisciplinary management of BC and adjuvant RT reduces local recurrences and has survival benefits.^17^ In our study, the rate of receiving adjuvant RT was significantly lower in Syrian patients and as an expected result, the rate of local recurrence was higher in this group.

 In our study, the OS of Syrian refugees was significantly inferior compared to Turkish patients (Figure 1). We also showed that receiving adjuvant CT, RT, and HT are independent risk factors associated with long survival. According to our study findings, this survival inferiority in refugees seems to be related to inadequately administered adjuvant treatments. However, other factors may have negatively affected the survival of Syrian patients. Most of Syrian refugees live in camps, and are more likely to develop infectious diseases and malnutrition. It was not possible to evaluate these issues in this retrospectively designed observational study based on hospital electronic records. However, it is clear that refugees have a high risk for these factors that may adversely affect cancer survival.

 In the survival comparison, by stratifying the two groups according to the stages, patients with locally advanced stage who benefited the most from adjuvant treatments showed the greatest difference between the two groups. The survival curve also showed a remarkable survival difference between the two groups in the early stage. Turkish patients also had better survival in this stage than Syrian patients (Figure 2). The small number of patients or the short follow-up period may be related to lack of statistical significance in this group. Since early-stage patients already have long survival, a long follow-up is required to show the OS difference. Patients with metastatic disease had the worst survival and there was no significant difference between the two groups. Numerically, the median OS was better in the Turkish group.

 In our study, the survival of groups from best to worst was as follows:

- Turkish patients with early-stage
- Turkish patients with locally advanced stage
- Syrian patients with early-stage
- Syrian patients with locally advanced stage.
- Turkish patients with metastatic disease
- Syrian patients with metastatic disease

Notably, Syrian patients with early stages had worse survival than Turkish patients with locally advanced stages. From an oncological point of view, this striking difference may reflect the inadequate treatments in Syrian patients, which is also supported by our study findings.

 One of the most important limitations of our study was the small sample size, especially in Syrian patients. However, in this observational study, we included all patients in our clinic who met the inclusion criteria during the study period. We are aware that the sample sizes are small for survival comparison, especially for subgroups according to stages. Nevertheless, we believe that presenting our data with the current number of patients and follow-up period will shed some light for more comprehensive studies to be conducted. Secondly, it was a retrospectively designed observational study and we could not evaluate the confounding factors that would affect the OS such as quality of life, nutritional status, or adherence to treatment. Lastly, there were unknown data including the initial stage of disease, histopathological features and treatments, particularly in patients diagnosed and treated in Syria. Especially in HER-2, which is a parameter requiring more equipped laboratories for evaluation, the unknown rate was high. The high number of unknown parameters in Syrian patients may be related to the difficulty of accessing medical services in the wartime conditions, as well as possibly the transfer problems during migration. We could not evaluate the indications of adjuvant treatments because of missing data. For this reason, we made a general comparison between the groups in terms of the applied treatments modalities.

 The strength of our study was the comparison of refugees with a demographically similar group living in a very close geography. In this study, which tried to determine how patients with BC were affected by the crisis, almost all Syrian patients were diagnosed after 2011 and this supports that our findings can reflect the crisis effect.

 In conclusion, Syrian refugees with BC are more likely to receive suboptimal or missing treatment that includes adjuvant CT, adjuvant RT, and adjuvant HT which have survival benefit. They have inferior survival compared to local patients, particularly in locally advanced diseases. Our findings highlight the need for the provision of cancer therapy for such vulnerable populations. We suggest that more attention should be paid to BC, as it is the most common cancer among refugees.
